# Prognosis of stage III colorectal carcinomas with FOLFOX adjuvant chemotherapy can be predicted by molecular subtype

**DOI:** 10.18632/oncotarget.17023

**Published:** 2017-04-11

**Authors:** Yujin Kwon, Minhee Park, Mi Jang, Seongju Yun, Won Kyu Kim, Sora Kim, Soonmyung Paik, Hyun Jung Lee, Sungpil Hong, Tae Il Kim, Byungsoh Min, Hoguen Kim

**Affiliations:** ^1^ Department of Pathology and BK21 for Medical Science, Yonsei University College of Medicine, Seoul, Korea; ^2^ Severance Biomedical Science Institute and BK21 for Medical Science, Yonsei University College of Medicine, Seoul, Korea; ^3^ Department of Internal Medicine, Yonsei University College of Medicine, Seoul, Korea; ^4^ Department of Surgery, Yonsei University College of Medicine, Seoul, Korea

**Keywords:** colon cancer, mRNA expression-based molecular classification, molecular subtype, consensus molecular subtype, DNA microarray

## Abstract

Individualizing adjuvant chemotherapy is important in patients with advanced colorectal cancers (CRCs), and the ability to identify molecular subtypes predictive of good prognosis for stage III CRCs after adjuvant chemotherapy could be highly beneficial. We performed microarray-based gene expression analysis on 101 fresh-frozen primary samples from patients with stage III CRCs treated with FOLFOX adjuvant chemotherapy and 35 matched non-neoplastic mucosal tissues. CRC samples were classified into four molecular subtypes using nonnegative matrix factorization, and for comparison, we also grouped CRC samples using the proposed consensus molecular subtypes (CMSs). Of the 101 cases, 80 were classified into a CMS group, which shows a 79% correlation between the CMS classification and our four molecular subtypes. We found that two of our subtypes showed significantly higher disease-free survival and overall survival than the others. Group 2, in particular, which showed no disease recurrence or death, was characterized by high microsatellite instability (MSI-H, 6/21), abundant mucin production (12/21), and right-sided location (12/21); this group strongly correlated with CMS1 (microsatellite instability immune type). We further identified the molecular characteristics of each group and selected 10 potential biomarker genes from each. When these were compared to the previously reported molecular classifier genes, we found that 31 out of 40 selected genes were matched with those previously reported. Our findings indicate that molecular classification can reveal specific molecular subtypes correlating with clinicopathologic features of CRCs and can have predictive value for the prognosis for stage III CRCs with FOLFOX adjuvant chemotherapy.

## INTRODUCTION

Colorectal carcinomas (CRCs) have variable clinical, pathologic, and molecular features. Currently, these are classified based on the histologic findings, and tumor staging is determined by assessing spread at the time of diagnosis. Accurately determining the prognosis for individual patients with CRC is important, both for disease management, and for patient life planning. At present, prognosis is based predominantly on the pathologic stage of disease. However, formulating accurate projections for patients with stages II and III cancer is difficult, due to the fact that these patients have intermediate survival rates, and predicting individual responses to adjuvant chemotherapy is currently impossible. For stage III CRCs, standard therapy involves curative surgery, followed by adjuvant chemotherapy with the FOLFOX regimen. However, no biomarkers or classification system for accurately determining prognoses after this chemotherapeutic regimen in stage III CRCs is available.

Gene expression-based subtyping is widely accepted as a relevant method for assessing disease stratification [1, 2]. Recently, an international consortium [3] was dedicated to network analysis of six previously published independent classifiers [2, 4–8] using Jaccard distance, Markov cluster (MCL) algorithm, and weighted silhouette width (R package ‘WeightedCluster’). Four consensus molecular subtypes (CMSs) of CRC were established with the following distinguishing features: CMS1 (microsatellite instability immune, 14%) indicative of hypermutations and microsatellite unstable features which generally accompany strong immune activation; CMS2 (canonical, 37%) showed epithelial characters and marked WNT and MYC signaling activation; CMS3 (metabolic, 13%) showed also epithelial characters and metabolic dysregulation; CMS4 (mesenchymal, 23%) showed stromal infiltration, strong angiogenic features, and hyperactivation of transforming growth factors (TGF-β). Samples with mixed features (13%) were interpreted as a transition phenotype or represent an intratumoral heterogeneity [3].

Although these molecular subtypes can predict clinicopathologic characteristics and the natural prognosis of the disease, no gene expression-based subtyping system is available for calculating the prognoses of stage III CRCs after FOLFOX chemotherapeutic regimen. Specifically, evaluating the response to FOLFOX adjuvant chemotherapy according to molecular subtype may provide insights into 1) the natural course of stage III CRCs after FOLFOX chemotherapy according to molecular subtypes, and 2) the identification of specific subgroup(s) that most benefit from FOLFOX chemotherapy.

In this study, we selectively used fresh-frozen tissues of stage III CRCs treated with FOLFOX adjuvant chemotherapy. Homogeneous tumor cell population (> 70% tumor cells) of each tissue was collected by microdissection technique, which allowed us to minimize experimental error caused by differential proportion of tumor cells in tissue samples. We analyzed mRNA expression profiles of stage III CRCs samples and matched non-neoplastic colon mucosal tissues. In doing so, we identified colon cancer-specific transcripts and further found that CRC gene expression profiles can be divided into four types by nonnegative matrix factorization analysis. Moreover, we demonstrated an association between our specific molecular subtypes and the CMS, and further identified subtypes associated with good prognosis for stage III CRCs after FOLFOX chemotherapy.

## RESULTS

### Unsupervised clustering analysis of mRNA expression profiles identifies four distinct molecular subtypes

**Figure 1:**
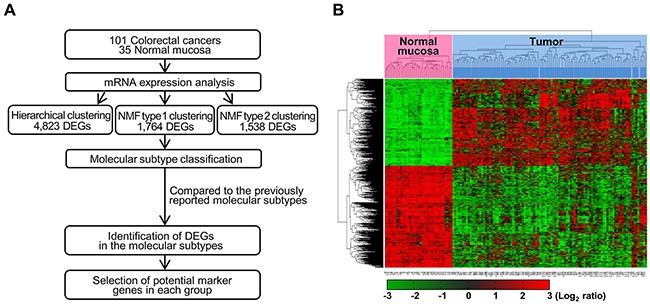
Workflow overview of gene expression-based molecular classification **(A)** NMF consensus clustering using 1,764 DEGs in tumors compared to non-neoplastic tissues. DEGs for each of the four groups, as compared to non-neoplastic colon mucosa tissues and/or the other groups were selected. DEG: differentially expressed genes, NMF: nonnegative matrix factorization. **(B)** Unsupervised hierarchical clustering analysis of mRNA expression profiles from colon cancer tissues. Unsupervised classification identifies four molecularly distinct subtypes. Red and green colors indicate transcript levels above and below the sample median, respectively. Complete separation of 35 normal colon tissues (red) and 101 colon cancers (sky blue) was evident based on gene expression profiles.

We first determined the mRNA expression profiles of 101 CRCs and 35 non-neoplastic colon mucosa tissue samples by microarray analysis, using the Human HT-12 v4 Expression BeadChip, which contains 47,323 probes representing 31,332 annotated genes (Figure 1A). As a result, 4,823 genes were identified as differentially expressed genes (DEGs) that displayed a |fold change|>2 and *P*-value<0.05 in colon cancers, as compared to non-neoplastic colon mucosa. To further analyze colon cancer-specific gene expression, we used unsupervised hierarchical clustering analysis of the 4,823 DEGs and found that CRC samples grouped into four separate clusters, distinct from the non-neoplastic colon mucosa tissues (Figure 1B). These gene expression microarray data have been deposited in NCBI's Gene Expression Omnibus (GEO) database (http://www.ncbi.nlm.nih.gov/geo/) and are accessible through the GEO Series accession number GSE83889.

### Gene expression-based molecular subtypes are categorized in four groups by nonnegative matrix factorization

**Figure 2:**
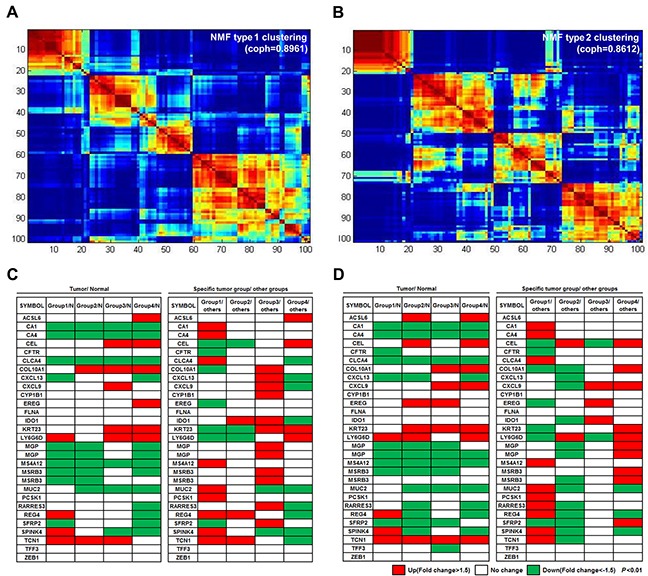
Four molecular subtypes identified using consensus clustering-based NMF **(A)** NMF type 1 clustering: consensus clustering using 1,764 DEGs in tumors compared to non-neoplastic tissues. **(B)** NMF type 2 clustering: clustering with 1,538 DEGs specific for one group, as compared to other tumor groups. **(C** and **D)** Analysis of the mRNA expression patterns of the 30 genes from the CRCassigner-30 was performed according to each of the four subtypes from NMF type 1 clustering **(C)** and NMF type 2 clustering **(D)**. NMF type 1 clustering showed more distinct gene expression patterns between the tumor subtypes than that of the NMF type 2 clustering.

To classify the 101 colorectal tumors according to the gene profiles of those tumors, we performed NMF consensus clustering using 1,764 tumor-specific DEGs that displayed a |fold change|>2 and *P*-value<0.01 in a more strict condition compared to non-neoplastic tissues. NMF is a nonnegative matrix factorization algorithm that focuses on the analysis of data matrices whose elements are nonnegative. To identify high consensus clusters (cophenetic coefficient, ρk, is closer to 1.0), we applied the various conditions for the factorization algorithm for each value of k cluster (k=2, 3, 4, 5, 6, and 7). We then obtained a consensus plot (and cophenetic coefficient) for each k=2 to 7 value and chose k values with high consensus. Using this consensus analysis with the 1,764 DEGs, we found good consensus for k=4 clusters (coph=0.8961), suggesting that there was evidence for four consensus clusters (type 1) and hence, four functional distinct properties that can be elucidated from the DEGs of 101 colorectal tumors; group 1 (n=22, 21.8%), group 2 (n=21, 20.8%), group 3 (n=16, 15.8%), and group 4 (n=42, 41.6%) (Figure 2A). The consensus matrix showed that groups 1 and 4 appeared as individualized clusters (sharp consensus clustering), whereas groups 2 and 3 showed less distinct boundaries.

We also performed NMF consensus clustering with another selected gene set; the 1,538 DEGs that showed differential expression among tumor groups were divided by the hierarchical clustering method (Figure 1B) when |fold change|>2 and *P*-value<0.01. In the NMF consensus analysis with these 1,538 DEGs, there are k=4 clusters (type 2) with high cophenetic coefficient (coph=0.8612); group 1 (n=20, 19.8%), group 2 (n=29, 28.7%), group 3 (n=24, 23.8%), and group 4 (n=28, 27.7%) (Figure 2B). Samples represented in each group divided by NMF with either the 1,764 or the 1,538 DEGs were similar (77.2% matched).

In order to further characterize the gene expression of the different tumor subtypes, we analyzed expression patterns of 30 colon cancer classifier genes, known as the CRCassigner-30 [2], in four subtypes that were obtained each from type 1 and type 2 clustering under the conditions of |fold change|>1.5 and *P*-value<0.01 (Figure 2C, 2D). In four tumor groups obtained from type 1 clustering, the expression of 5 genes was found to have increased in a specific tumor group, compared to the normal group (left panel of Figure 2C). In addition, the expression of 18 genes was found to have increased in a specific tumor group, compared to the rest of the tumor groups (right panel of Figure 2C). In four tumor groups obtained from type 2 clustering, the expression of two and 17 genes was also found to have increased in a specific tumor group compared to the normal group or the rest of the tumor groups, respectively (Figure 2D). Overall, type 1 clustering (NMF with 1,764 DEGs in tumors compared to non-neoplastic tissues) showed more distinct gene expression patterns between tumor groups than those of type 2 clustering (NMF with 1,538 DEGs in tumor groups divided by unsupervised hierarchical clustering). Therefore, we chose type 1 clustering for the subsequent gene expression analyses.

### Clinicopathologic characteristics of CRCs according to molecular subtypes

We compared several clinicopathologic and molecular characteristics of our 101 CRCs according to the four molecular subtypes; the clinicopathologic characteristics of the samples in each of the four molecular subtypes are shown in Table 1. We found that there were several clinicopathologic parameters, showing significant differences in each group. Specifically, there are significant differences among the subtypes in tumor location (*P*<0.001), proportion of microsatellite instability (MSI) (*P*<0.001), presence of mucin (*P*<0.001), and incidence of *KRAS* mutation (*P*=0.01). Further, >50% of the cases in groups 3 (62.5%) and 4 (85.7%) presented as left-sided tumors. Conversely, >50% of the cases in groups 1 (54.5%) and 2 (57.1%) were located on the right side. Histologically, most cases were adenocarcinomas with moderate differentiation; there were four mucinous adenocarcinomas (mucin production in >50% of tumors) and two medullary carcinomas (carcinoma with prominent infiltration of intraepithelial lymphocytes). All four mucinous adenocarcinomas were in group 1, and the two medullary carcinomas were in group 2. In regards to extracellular mucin production, most cases in groups 3 (87.5%) and 4 (92.9%) did not present with extracellular mucin production; whereas, >50% of cases in groups 1 and 2 showed extracellular mucin. We also found frequent *KRAS* mutations in group 1 (63.6%) and group 3 (56.3%) and less in groups 2 (28.6%) and 4 (26.2%). Further, we observed high MSI (MSI-H) in groups 2 (28.6%), and 1 (9%), but no instances in groups 3 and 4.

**Table 1:** Clinical and pathologic features of each molecular group

Category	Variables	Group 1		Group 2		Group 3		Group 4		*P*-value
		(n=22, 21.8%)		(n=21, 20.8%)		(n=16, 15.8%)		(n=42, 41.6%)		
Age, years		57.95±13.26		58.09±13.67		62±9.04		58.81±9.42		0.802
Gender	Male	14	63.6%	8	38.1%	7	43.8%	22	52.4%	0.376
	Female	8	36.4%	13	61.9%	9	56.2%	20	47.6%	
Location	Right side	12	54.5%	12	57.1%	6	37.5%	6	14.3%	<0.001^a^
	Left side	10	45.5%	9	42.9%	10	62.5%	36	85.7%	
Preop CEA level	≤5 ng/ml	18	81.8%	17	81.0%	10	62.5%	28	66.7%	0.367
	>5 ng/ml	4	18.2%	4	19.0%	6	37.5%	14	33.3%	
Histologic diagnosis	Adenocarcinoma	18	81.8%	19	90.5%	16	100.0%	42	100.0%	<0.001^a^
	Mucinous adenocarcinoma	4	18.2%	0	0.0%	0	0.0%	0	0.0%	
	Medullary carcinoma	0	0.0%	2	9.5%	0	0.0%	0	0.0%	
Mucin formation	Absent	10	45.5%	9	42.8%	14	87.5%	39	92.9%	0^a^
	Focal	8	36.4%	12	57.2%	2	12.5%	3	7.1%	
	predominant	4	18.2%	0	0.0%	0	0.0%	0	0.0%	
Crohn like lymphoid reaction	low-density group	14	63.6%	13	61.9%	8	50.0%	33	78.6%	0.157
high-density group		8	36.4%	8	38.1%	8	50.0%	9	21.4%	
invasion pattern	expending	2	9.1%	7	33.3%	2	12.5%	4	9.5%	0.098
	infiltrative	20	90.9%	14	66.7%	14	87.5%	38	90.5%	
*BRAF*^b^	Wild type	22	100.0%	20	95.2%	16	100.0%	42	100.0%	0.366
	Mutation	0	0.0%	1	4.8%	0	0.0%	0	0.0%	
*KRAS*^b^	Wild type	8	36.4%	15	71.4%	7	43.8%	31	73.8%	0.01^a^
	Mutation	14	63.6%	6	28.6%	9	56.3%	11	26.2%	
MSI status	MSS/ MSI-low	20	90.9%	15	71.4%	16	100.0%	42	100.0%	<0.001^a^
	MSI-high	2	9.0%	6	28.6%	0	0.0%	0	0.0%	
	MLH1 loss^c^	0		6		0		0		
	MSH2 loss^c^	1		1		0		0		
	PMS2 loss^c^	1		5		0		0		
	MSH6 loss^c^	1		1		0		0		

^a^*P*<0.05, ^b^Pyrosequencing, ^c^Immunohistochemical stain, MSS: Microsatellite stability, MSI: Microsatellite instability

Protein expression of the mismatch repair genes (MLH1, MSH2, PMS2, and MSH6) were then evaluated by immunohistochemistry. All six cases of MSI-H in group 2 showed loss of expression of MLH1 (five cases showed loss of MLH1 and PMS2 expression, and the remaining one case showed loss of MLH1, PMS2, and MSH6 expression). In contrast, one MSI-H case in group 1 showed loss of expression of PMS2, and the other case showed loss expression of MSH2 and MSH6. One out of four mucinous adenocarcinomas was MSI-H, and both medullary carcinomas were MSI-H. Crohn's-like lymphoid reaction is a common feature of MSI in colon cancer, and therefore, this was evaluated using the Väyrynen-Mäkinen criteria. However, no significant differences were found between the different subtypes.

### Correlation between our four molecular subtypes and the consensus molecular subtypes

We next employed ‘CMSclassifier’ (scripts and code for CMS classifier available at: https://github.com/Sage-Bionetworks/crcsc) for CMS classification [2, 3] of our 101 CRC samples using k=4 clusters determined in Figure 2A. Among the 101 samples, 80 tumors were representative of each CMS; whereas, the remaining 21 unlabeled samples (non-consensus samples) did not have any consistent pattern within the four CMS groups. When we compared the CMS grouping with our subtypes, and found that, of the 101 cancers, 63 showed close correlation to one of the four subtypes (Table 2). Specifically, 10 of 21 group 2 samples were included in CMS1 (microsatellite instability immune and characterized as hypermutated, microsatellite unstable and strong immune activation), and all 10 that grouped in CMS1 were also classified in group 2. Both the group 2 and CMS1 samples were characterized by frequent MSI-H tumors. Similarly, 31 out of 42 samples from group 4 were classified as CMS2 (canonical and characterized by marked WNT and MYC signaling activation), while 31 out of 38 samples in CMS2 also were found in group 4. These group 4 and CMS2 types are characterized by frequent left-sided preponderance. We further observed that 12 out of 22 group 1 samples were classified as CMS3 (metabolic and characterized by evident metabolic dysregulation), and 12 out of 13 CMS3 cases belonged to group 1. In our study, 13 (13%) of CMS3 cases were characterized by high *KRAS* mutation rates. Finally, we found that 10 out of 16 samples belonging to group 3 were classified into CMS4 (mesenchymal and characterized by prominent transforming growth factor activation, stromal invasion and angiogenesis), while 10 out of 19 CMS4 samples also belonged to group 3. When we exclude the 21 non-consensus cases, a 79% (63 out of 80 cases) correlation was found between the CMS classification and our molecular classification. The distribution of the CMS groups is shown in Table 2.

**Table 2:** Correlation between 4 molecular groups and CMS subtypes

		NMF network class				Total
		Group 2	Group 4	Group 1	Group 3	
**Predicted CMS class**	CMS1	10 (66.6%)	0 (0%)	0 (0%)	0 (0%)	10
	CMS2	4 (26.7%)	31 (79.5%)	3 (18.8%)	0 (0%)	38
	CMS3	1 (6.7%)	0 (0%)	12 (75%)	0 (0%)	13
	CMS4	0 (0%)	8 (20.5%)	1 (6.2%)	10 (100%)	19
	undetermined	6	3	6	6	21
	Total	21	42	22	16	101

To evaluate how well NMF consensus clustering matched CMS classification, we further performed subtyping analysis by using the same set of samples used for NMF consensus clustering by another commonly used subtyping method that combines Euclidean distance, Ward's minimum variance method, and weighted gene co-expression network analysis (WGCNA). This analysis showed that 101 CRCs were divided into four main groups (group A-D), and all groups well-matched only CMS2 and not the remaining CMS subtypes (Supplementary Table 1). With the highest concordance rates, 13 out of 38 samples of group A, 14 out of 26 samples of group B, and half of 10 samples of group C belonged to CMS2. In group D, 6 out of 27 samples belonged to CMS2 or CMS4, respectively. These results showed that NMF consensus clustering was a highly compatible classification method to CMS classification.

We further compared several clinicopathologic and molecular characteristics of our 101 CRCs according to the CMS molecular subtypes. The clinicopathologic characteristics of four CMS molecular subtypes and non-consensus subtypes are shown in Supplementary Table 2. As expected, similar clinicopathologic parameters were found to vary according to both our classification system and the CMS grouping. There are significant differences among the subtypes in tumor location (*P*<0.01), proportion of MSI-H tumors (*P*<0.001), presence of mucin (*P*<0.001), and incidence of *KRAS* mutation (*P*<0.05). Cases classified as CMS1 displayed a right-sided preponderance (>70%), exophytic growth pattern, normal preoperative CEA level, and high preponderance of MSI-H tumors. In contrast, CMS2 cases a showed left-sided preponderance (84.2%) and frequent (31.6%) increased preoperative CEA level. Additionally, >50% of cases in CMS3 showed extracellular mucin production and frequent (61.5%) *KRAS* mutation.

### Patient survival according to molecular subtype

**Figure 3:**
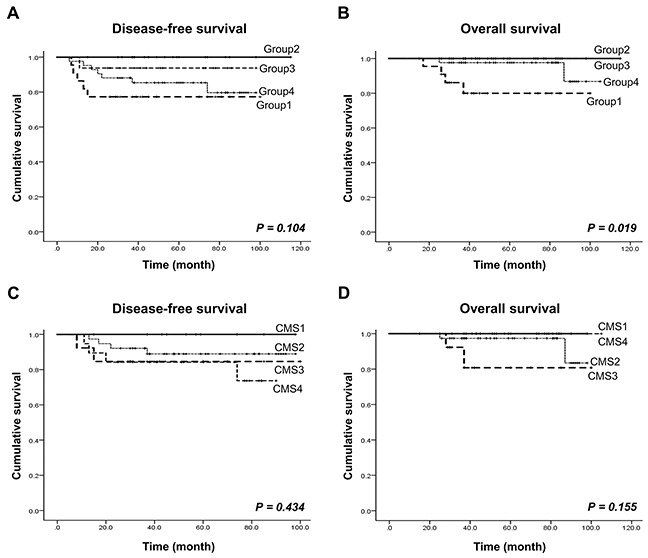
Patient survival rate analysis according to our CRC molecular subtype and the CMS **(A** and **B)** Disease-free survival and overall survival according to our four molecular subtypes. **(C** and **D)** Disease-free survival and overall survival according to the four CMS subtypes.

We performed a survival analysis on the 101 CRC patients according to the molecular subtypes. The mean follow-up time was 57.8 months (ranging from 15 to 115 months). With follow up, 13 patients (12.9%) relapsed, and six (5.9%) died from colon cancer-related causes. Groups 2 and 3 showed significantly better overall survival (OS) (*P*=0.019) than groups 1 and 4, which indicates that group 2 and 3 are associated with favorable prognosis (Figure 3A, 3B). We further performed survival analysis using a separate set of group 1, 2, and 3, and another set of group 2, 3, and 4, respectively. Groups 2 and 3 showed better disease-free survival (DFS) (*P*=0.039) and OS (*P*=0.027) than group 1, but not group 4 (Supplementary Figure 1). In consistent with these findings, the two subtypes from the CMS classification showing high concordance rates in overall survival were CMS1 and CMS4, which correspond to our groups 2 and 3, respectively (Figure 3C, 3D).

### Identification of differentially expressed genes in each molecular subtype

**Figure 4:**
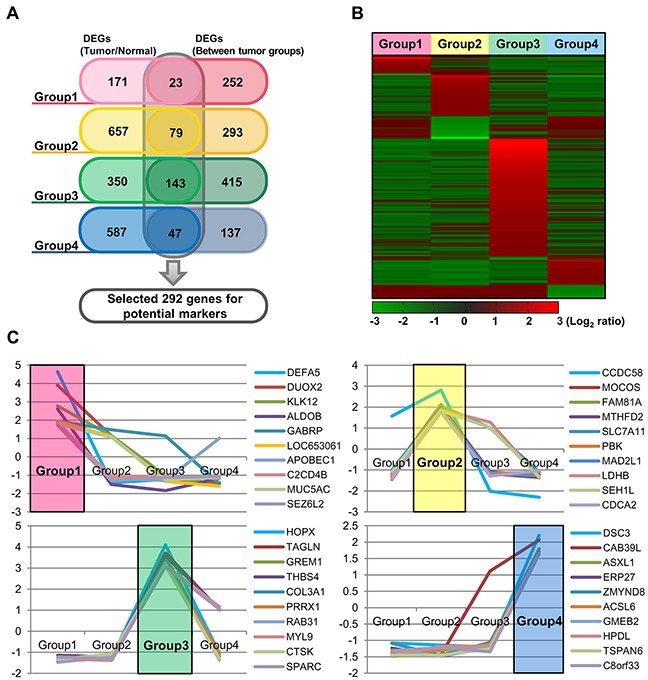
Identification of mRNAs differentially expressed in each of the four groups, as compared to non-neoplastic colon mucosa tissues and/or the other groups **(A)** Venn diagram showing a total of 292 potential marker genes, with group specific upregulation. **(B)** Heatmap of the mRNA expression profiles of these 292 DEGs according to the four molecular groups. **(C)** The mRNA expression profiles of 10 selected genes from each group.

We further analyzed gene expression patterns, in order to identify gene subsets that are differentially expressed in the four groups, as compared to non-neoplasctic colon mucosa tissues and/or other groups. Differently expressed genes were identified in each group: 194 genes in group 1,736 genes in group 2,493 genes in group 3, and 634 genes in group 4. In addition, 275 genes, 372 genes, 558 genes, and 184 genes were differentially expressed in each respective group, as compared to the other groups (Figure 4A). We next selected genes that are characteristic of each group by choosing those that are differentially regulated as compared to both normal tissue and the other molecular groups. As a result, 292 genes were found as potential group markers (Figure 4A, 4B). We finally selected ten genes from this group genes that are overexpressed >1.5-fold in one group, as compared to both normal tissues and tumors in other groups. Among these genes, especially, *DEFA5, DUOX2, KLK12*, and *ALDOB* in group 1, *CCDC58, MOCOS*, and *FAM81A* in group 2, *HOPX, TAGLN, GREM1, THBS4, COL3A1, PRRX1, RAB31, MYL9, CTSK*, and *SPARC* in group 3, and *DSC3* and *CAB39L* in group 4 were up-regulated (>2.0-fold) (Figure 4C, Table 3).

**Table 3:** Specific-upregulated genes in each molecular group

Gene cluster	Gene symbol^a^	Description	FC. T vs. N	FC. Specific T group vs. other T groups
**Group 1**	**DEFA5**	defensin, alpha 5, Paneth cell-specific	4.56	4.65
	**DUOX2**	dual oxidase 2	4.28	3.91
	**KLK12**	kallikrein-related peptidase 12	3.19	2.77
	ALDOB	aldolase B	5.37	2.61
	**GABRP**	gamma-aminobutyric acid (GABA) A receptor, pi	1.84	1.95
	LOC653061	similar to Golgin subfamily A member 8-like protein 1	2.62	1.95
	**APOBEC1**	apolipoprotein B mRNA editing enzyme, catalytic polypeptide 1	3.31	1.88
	C2CD4B	family with sequence similarity 148, member B	2.93	1.82
	**MUC5AC**	mucin 5AC, oligomeric mucus/gel-forming; similar to hCG1778310	2.27	1.79
	SEZ6L2	seizure related 6 homolog (mouse)-like 2	2.35	1.73
**Group 2**	CCDC58	coiled-coil domain containing 58	6.19	2.81
	**MOCOS**	molybdenum cofactor sulfurase	2.92	2.11
	FAM81A	family with sequence similarity 81, member A	2.59	2.06
	MTHFD2	methylenetetrahydrofolate dehydrogenase (NADP+ dependent)2, methenyltetrahydrofolate cyclohydrolase	3.10	1.99
	**SLC7A11**	solute carrier family 7, (cationic amino acid transporter, y+ system) member 11	2.52	1.93
	**PBK**	PDZ binding kinase	2.78	1.91
	MAD2L1	MAD2 mitotic arrest deficient-like 1 (yeast)	3.49	1.90
	LDHB	lactate dehydrogenase B	3.18	1.86
	SEH1L	SEH1-like (S. cerevisiae)	2.14	1.83
	CDCA2	cell division cycle associated 2	2.38	1.77
**Group 3**	**HOPX**	HOP homeobox	4.26	4.11
	**TAGLN**	Transgelin	2.66	3.73
	**GREM1**	gremlin 1	2.12	3.59
	**THBS4**	thrombospondin 4	2.85	3.58
	**COL3A1**	collagen, type III, alpha 1	5.33	3.34
	**PRRX1**	paired related homeobox 1	4.29	3.23
	**RAB31**	RAB31, member RAS oncogene family	3.60	3.21
	**MYL9**	myosin, light chain 9, regulatory	2.38	3.13
	**CTSK**	cathepsin K	2.59	3.11
	**SPARC**	secreted protein, acidic, cysteine-rich (osteonectin)	2.35	3.05
**Group 4**	DSC3	desmocollin 3	2.66	2.21
	**CAB39L**	calcium binding protein 39-like	2.22	2.07
	**ASXL1**	additional sex combs like 1 (Drosophila)	1.99	1.80
	**ERP27**	endoplasmic reticulum protein 27	2.55	1.70
	ZMYND8	zinc finger, MYND-type containing 8	1.90	1.69
	**ACSL6**	acyl-CoA synthetase long-chain family member 6	1.94	1.67
	GMEB2	glucocorticoid modulatory element binding protein 2	1.78	1.66
	HPDL	4-hydroxyphenylpyruvate dioxygenase-like	2.79	1.66
	**TSPAN6**	tetraspanin 6	2.11	1.64
	C8orf33	chromosome 8 open reading frame 33	2.15	1.63

^a^Selected genes that show specific-upregulated expression in each molecular group. Those matching previously reported classifier genes are marked in bold.

We next performed survival analysis according to the expression of two randomly selected genes out of 10 genes that represented characteristic of each group. Although statistically insignificant in some genes, there was a trend where cases were distinctively divided into poor and good prognosis groups according to the expression of selected gene (Supplementary Figure 2). High expression of *KLK12* and *MUC5AC* (group 1), and *DSC3* (group 4) was associated with poor prognosis, while high expression of *FAM81A*, *SEH1L*, *TAGLN* and *THBS4* (group 2 and 3), and *TSPAN6* (group 4) was associated with good prognosis.

## DISCUSSION

The identification of predictive biomarkers and the development of a molecular classification that can predict therapeutic responses of advanced CRCs are major goals in cancer research. A number of recent studies have elucidated several molecular CRC subtypes by gene expression profiling, and some of these have been reported to be associated with specific clinical outcomes [2, 4–8]. For example, Budinska *et al*. [4] divided CRCs into five subtypes, and demonstrated that one was associated with good outcomes (designated as lower crypt subtype) and another with poor outcomes (designated as mesenchymal subtype). Marisa *et al*. [5] outlined six CRC subtypes and found that one (designated as cancer stem cell and CIN normal subtype) was related to poor prognosis. Roepman *et al*. [7] proposed three subtypes, one of which (designated as the A subtype) was characterized by right-sided preponderance, was hypermutated with frequent MSI and *BRAF* mutations, and was associated with a good prognosis. De Sousa *et al*. [6] also divided CRCs into three molecular subtypes and found one (designated as CCS3 subtype) was related to poor differentiation and associated with poor prognosis. Sadanandam *et al*. [2] proposed five molecular subtypes, one of which (designated as stem-like subtype) was related to poor prognosis, and similarly, Schlicker *et al*. [8] identified five CRC subtypes, and found one (designated as 1.1 subtype) was related to poor prognosis and another (designated as 2.1 subtype) that was associated with better DFS. To comprehensively understand molecular CRC subtypes, we further matched these independently reported CRC subtypes to CMS subtypes. In Budinska *et al*.'s study [4], lower crypt subtype was associated with good outcomes, CIMP subtype was associated with worse survival after relapse, and mesenchymal subtype with worse relapse-free survival and overall survival. These three subtypes corresponded to CMS2, CMS1, and CMS4, respectively. Other subtypes such as cancer stem cell subtype (Marisa *et al*.) [5], C subtype (Roepman *et al*.) [7], CCS3 subtype (De Sousa *et al*.) [6], stem-like subtype (Sadanandam *et al*.) [2], and 1.1 subtype (Schlicker *et al*.) [8] were commonly related to worse relapse-free survival and overall survival, and these subtypes generally matched CMS4. These previously reported molecular subtypes were also associated with the characteristics of our groups. The *KRAS*-mutated subtype [5] is related to our group 1, whereas our group 2 is similar to the dMMR [5], A type [7] and CCS2 [6] subtypes, all of which are more commonly located in the right side of the colon and contain high MSI [9]. We further observed that our group 3 shares characteristics with the 1.3 subtype [8], which contains microsatellite stability (MSS) and activated transporter genes, and further, group 4 is associated with the B subtype [7], which is most commonly found in the left side of the colon.

The reported molecular classifications all have potential for both prognosis evaluation and for predicting the therapeutic responses of advanced CRCs. However, we believe that the previously reported molecular classification systems have several features limiting their clinical utility. Specifically, many of the published studies have a diverse clinical cohort, as well as non-uniform sources for clinical sample acquisition (e.g., RNA extraction from fresh-frozen samples or from formalin-fixed paraffin-embedded samples). Further, experimental methodological differences and distinct data processing algorithms may produce discrepant results. Therefore, in order to establish a collaborative subtyping for CRC and to resolve inconsistencies among the reported gene expression-based CRCs, four CMSs with distinguishing features were proposed by an international consortium [3]. By this CMS classification, 80 out of 101 cases analyzed in our study were classified into one of the four groups, whereas 21 cases were not categorized and thus placed in the non-consensus group. Here, we performed NMF consensus clustering with 1,764 DEGs in tumors compared to non-neoplastic tissues and identified four CRC molecular subtypes. We found close correlation between group 2 and CMS1 (microsatellite instability immune), group 4 and CMS2 (canonical), group 1 and CMS3 (metabolic), and group 3 and CMS4 (mesenchymal).

Although each of our molecular subtypes generally matched to a specific CMS, and they showed similarities to the CMS system, there are also some critical differences. Specifically, our molecular grouping method divided all 101 CRC cases into one of the four groups, whereas 21 cases were not categorized using the CMS system. Of the remaining 80 cases that were classified by the CMS, 79% were grouped in a correlating subtype. Therefore, 63% of the 101 cases were completely matched in both systems. Since we evaluated the predictive value of molecular classification using only stage III CRCs with FOLFOX adjuvant chemotherapy and performed microdissection of each fresh-frozen sample, we believe that our approach enabled the analysis of homogeneous tumor cell populations (>70% tumor cells); therefore, experimental errors from different tumor populations were minimalized. We rather suspect that the discrepant results might result from the diverse patient cohorts, sample preparation methods, and different technologies utilized for the analysis of gene expression platforms.

Our NMF consensus clustering-based molecular classification showed that patients in groups 2 and 3 exhibited a significant advantage in overall survival (*P*=0.019), as well as a trend toward better DFS (*P*=0.104). More specifically, survival analysis using groups 1, 2, and 3 revealed that patients in groups 2 and 3 showed better DFS (*P*=0.039) and OS (*P*=0.027) than group 1, but no significant difference in survival was observed when we analyzed groups 2, 3 and 4. Supporting these findings, a previous report showed that stages II and III CRC patients with good prognosis could be categorized into a subtype highly similar to group 2, with features such as MSI, *BRAF* mutations, and right-sided tumor location [5–7]. Moreover, the overall survival curve for our patients based on CMS subtype showed a worse outcome for CMS2 and CMS3, which correlate to groups 4 and 1, respectively, in our study. However, statistical significance was not found in the CMS classification, likely due to small number of cases that were classified. These findings suggest that our molecular classification can identify homogeneous subsets of stage III CRCs with either a good or bad prognosis after FOLFOX adjuvant chemotherapy. We note, however, that we could not conclude that the clinical results from the patients with good prognosis subtypes can predict responses to the FOLFOX regimen or the natural clinical disease course, as this was not a prospective study. To better predict responses to FOLFOX adjuvant therapy, a large cohort including CRC mucosa tissues without FOLFOX adjuvant chemotherapy should be included in future study.

We analyzed the molecular characteristics of each subtype by performing gene ontology analysis with the differentially expressed subtype-specific genes. This revealed that the expression of cell death and apoptosis genes were lower in group 1 tumors. It has previously been reported that the important functional consequence of CRCs with a *KRAS* mutation is lack of apoptosis, and 14 out of 22 CRCs in group 1 had a *KRAS* mutation [10, 11]. In group 2, increased expression of genes associated with p53 signaling and cell proliferation was noted, and six out of eight MSI-H CRCs were classified in this group. MSI-H tumors are known to have a more favorable prognosis than the MSS/MSI-L CRCs. Further, it has been reported that increased expression of p53 is associated with good survival outcome in the stage III CRCs with FOLFOX therapy [12]. Accordingly, the group 2 CRCs in our study showed the best survival after FOLFOX therapy. In group 3 CRCs, expression of genes associated with inhibition of epithelial-to-mesenchymal transition was increased, whereas genes associated with fatty acid synthesis and the TCA cycle were decreased. The association between decreased expression of metabolic genes and good prognosis had been reported in both CRC and hepatocellular carcinoma [13, 14], and agreement with this, CRC patients in group 3 showed a better prognosis than those in the other groups. In group 4 CRCs, decreased expression of genes associated with antigen processing and presentation and immune response was observed, and this has previously been associated with a poor prognosis in many cancers [15–21]. In agreement with this, the prognosis for group 4 CRCs was the worst of any of the groups in our study.

We further selected 10 potential biomarker genes from each group by identifying the genes that were differentially expressed both between the tumor groups and normal mucosa. We then compared these genes to those selected as a molecular classifier in a number of previously published papers [2, 4–8]. We found that >50% of our genes matched previously reported classifier genes (comprised of a total of 2032 genes from six different studies). In group 1, six genes [*DEFA5* (4.56/4.65-fold), *DUOX2* (4.28/3.91-fold), *KLK12* (3.19/2.77-fold), *GABRP* (1.84/1.95-fold), *APOBEC1* (3.31/1.88-fold), and *MUC5AC* (2.27/1.79-fold)] matched previously reported genes. Of these, *DEFA5* had been shown to be a key factor in the formation of adenomas, and has been proposed as a prognostic and predictive potential molecular biomarker in CRCs [22]. In group 2, three genes [*MOCOS* (2.92/2.11-fold), *SLC7A11* (2.52/1.93-fold) and *PBK* (2.78/1.91-fold)] matched previously reported genes. The over-expression of *SLC7A11*, a p53-associated gene, had been shown to have an important role in tumor growth suppression [23]. In group 3 CRCs, all 10 genes (*HOPX, TAGLN, GREM1, THBS4, COL3A1, PRRX1, RAB31, MYL9, CTSK* and *SPARC*) matched the previously reported molecular classifier [2, 4–8], and most of these genes were known to function as suppressors during EMT process. *HOPX* (4.26/4.11-fold) is a known colon stem cell marker and is associated with the suppression of tumor metastasis [24], and *TAGLN* (2.66/3.73-fold) was found as a novel tumor suppressor and its post-surgical high expression was reported to be associated with good prognosis in stage III CRCs [25]. *GREM1* (2.12/3.59-fold) was reported to be closely associated with low lymphovascular invasion and good prognosis in locally advanced stage II and III CRCs [26]. In group 4, five genes genes [*CAB39L* (2.22/2.07-fold), *ASXL1* (1.99/1.80-fold), *ERP27* (2.55/1.70-fold), *ACSL6* (1.94/1.67-fold), and *TSPAN6* (2.11/1.64-fold)] matched previously reported classifier genes, and of these, *TSPAN6* was the most frequently reported in the six previous papers. *TSPAN6* has been associated with tumor cell migration [27, 28], and over-expression is linked to worsened survival in CRC patients. We further matched our potential biomarker genes to classifier genes provided from a recently reported study, which merged and evaluated 22 types of previously reported classifiers [29]. Overall, about 80 % of our potential biomarker genes overlapped with classifier genes reported by 22 different previous studies. These findings indicate that our genes may serve as clinically useful molecular biomarkers for tumor classification.

Collectively, these findings suggest that molecular classification using these molecular markers may be possible, and future prospective studies may further elucidate their prognostic and predictive values for assessing the response to FOLFOX adjuvant therapy in stage III CRCs.

## MATERIALS AND METHODS

### Patients and tumor tissues

A total of 101 CRCs and 35 matched non-neoplastic colon mucosal tissue samples were analyzed in this study. All the cancers were stage III (metastasis to regional lymph nodes but not to distant sites) and were treated with the FOLFOX regimen; none of the patients had received neo-adjuvant chemotherapy. The specimens were obtained from the archives of the Department of Pathology, Yonsei University, Seoul, Korea and from the Liver Cancer Specimen Bank of the National Research Resource Bank Program of the Korean Science and Engineering Foundation of the Ministry of Science and Technology. Information from the tumor registry and chart review data were obtained to determine demographics, tumor site, and follow up. The population contained 50 females and 51 males. The 36 tumors located proximal to the splenic flexure were classified as right-sided and the 65 distal to the splenic flexure as left-sided. Patient data were collected retrospectively. All patients had undergone curative colorectal resection between 2006 and 2012, and fresh snap-frozen samples were obtained immediately at the time of surgery. Tumor specimens were microdissected using a cryostat and fractionated to improve tumor content. Briefly, prior to cutting sections for RNA isolation, a slide was prepared for hematoxylin-eosin staining to allow the selection of samples with >70% tumor cells; samples with a tumor cell content <70% were further cut to enrich the tumor cell population. Through this process, were ensured that all carcinoma samples analyzed in this study were comprised of >70% tumor cells. Authorization for use of these tissues for research purposes was obtained from the Institutional Review Board of the Yonsei University of College of Medicine.

### RNA preparation

Total RNA was extracted using TRIzol Reagent (Invitrogen Life Technologies, Carlsbad, CA, USA), according to the manufacturer's protocol. After DNase digestion and other clean-up procedures, RNA samples were quantified, aliquoted, and stored at −80°C until use. For quality control, RNA purity and integrity were evaluated by denaturing gel electrophoresis and measurement of the A260/280 ratio, and all samples were analyzed using the 2100 Bioanalyzer (Agilent Technologies, Santa Clara, CA, USA). For all samples, the RNA integrity number scores were >9.5.

### Gene expression analysis

For DNA microarray hybridization, RNA was pooled by mixing equal amounts of total RNA, and biotin-labeled cRNA targets were synthesized starting from 1.5 μg of total RNA. Double-stranded cDNA synthesis was performed using the Illumina® TotalPrep RNA Amplification Kit (Illumina, San Diego, CA, USA), while biotin-UTP-labeled antisense RNA was transcribed *in vitro* using the Ambion MEGAscript kit (Ambion Life Technologies, Carlsbad, CA, USA). All steps of the labeling procedure were performed according to the manufacturers' protocols. Microarray experiments were conducted on the HumanHT-12 v4 Sentrix Expression BeadChip (Illumina), which contains 47,231 probes, representing 31,332 annotated genes. Hybridization of labeled cRNA to the BeadChip, washing, and scanning were performed according to the Illumina Bead Station 500× manual.

### mRNA gene expression data preparation and statistical analysis

Raw data were extracted using the software provided by the manufacturer (Illumina Genome Studio v2011.1 [Gene Expression Module v1.9.0]), and expression intensities were normalized using quantile normalization techniques [30]. Based on these normalized intensities, genes differentially expressed in non-neoplastic colon mucosal tissues and in colon tumors were determined using the integrated statistical method previously reported [31]. Briefly, 1) two independent tests were performed: a Student's *t*-test and the log_2_-median-ratio test; 2) adjusted *P*-values from each test were computed using an empirical distribution of the null hypothesis that the means of the genes are not different, which was obtained from random permutations of the samples; 3) the *P*-values from the two tests were combined to compute the overall *P*-values using Stouffer's method [32], and 4) For unsupervised hierarchical clustering in Figure 1B, 4,823 DEGs were selected from the microarray raw data under the conditions of |fold change|>2 and *P*-value<0.05. For NMF consensus clustering analysis in Figure 2A, 1,764 DEGs were selected from the microarray raw data under the conditions of |fold change|>2 and *P*-value<0.01. For NMF consensus clustering analysis in Figure 2B, 1,538 DEGs were selected from DEGs among tumor groups that were divided by unsupervised hierarchical clustering under the conditions of |fold change|>2 and *P*-value<0.01. For gene expression analysis in Figure 2C and 2D, |fold change|>1.5 and *P*-value<0.01 were applied to obtain DEGs belonging to CRCassigner-30, which was a group of 30 genes with high scores calculated by PAM (nearest shrunken centroids–based method) [33] reported in a previous study [2]. Finally, functional enrichment analysis of the differentially expressed genes was performed using DAVID software [34], in order to identify GO biological processes and Kyoto Encyclopedia of Genes and Genomes (KEGG) pathways represented by the genes in individual clusters with statistical significance.

### Molecular subtyping and comparison to published consensus molecular subtype

For molecular subtyping, we applied a hierarchical clustering and nonnegative matrix factorization (NMF) [35–37], using the gene expression profiles from CRC tissues. In brief, NMF consensus clustering method resorts to factor the gene-expression matrix *A* into the product of two matrices of positive entries, *A ∼ WH*. Matrix *W* has size *N × k* and Matrix *H* has size *k × M*. *k* is much smaller than *M*. The column of *W* defines a ‘metagene’, with entry *wij* the coefficient of gene *i* in metagene *j*. The columns of Matrix *H* represent metagene expression pattern of the corresponding sample, with each entry *hij* representing the expression level of metagene *i* in sample *j*. Given factorization of *A ∼ WH*, Matrix *H* can be used to determine the cluster membership: sample *j* is placed in cluster *i* if the *hij* is the largest entry in column *j* [35]. Differentially expressed genes were selected using complete linkage for hierarchical clustering based on the Pearson coefficient correlation algorithm, and these were utilized for NMF with MATLAB software. Molecular subtyping was performed with consensus clustering-based NMF, according to the optimal number of clusters, which was determined based on the cophenetic correlation coefficient (coph) values [37, 38] determined in our previous study (coph=0.8961). We performed a repeated NMF clustering analysis using the same set of samples with 1,764 DEGs under the same condition, and confirmed that the four groups divided by type 1 clustering were reproducible (93% matched). To subtype our samples by another method, we adopted Ward's minimum variance method in combination with Euclidean distance and weighted gene co-expression network analysis (WGCNA) [39]. WGCNA was used to verify the quality of our microarray data, and Ward's minimum variance method according to Euclidean distance was used to cluster 101 colorectal tumors. To compare our molecular subtypes with the previously reported CMSs, we used CMSclassifier [3], which includes the random forest classifier, as well as a ‘single-sample predictor’ (SSP) classifier. Further, the prognosis predicting values of our molecular subtypes and CMS subtypes were compared and analyzed by ‘CMSclassifier’.

### *KRAS* and *BRAF* mutation analysis

*KRAS* mutation analysis was performed via pyrosequencing, using the CE-IVD Marked PyroMark *KRAS* Kit (QIAGEN, Hilden, Germany), according to the manufacture's protocols (Therascreen *KRAS* Pyro Kit Handbook, version 1, July 2011). For each sample, 10 ng of genomic DNA was used for analysis of mutations in codons 12 and 13 and another 10 ng DNA was utilized to identify mutations in codon 61. Pyrosequencing was also employed for *BRAF* mutation analysi*s* to detect the *BRAF* V600E mutation, as previously reported [40].

### Immunohistochemistry

Paraffin-embedded tissue blocks were cut into 4-μm sections. Immunohistochemical analysis was performed using a Ventana XT automated stainer (Ventana Corporation, Tucson, AZ, USA) with antibodies against the following: MutL homolog 1 (MLH1, diluted 1:50, BD Biosciences, San Jose, CA, USA), MutS homolog 2 (MSH2, diluted 1:200, BD Biosciences), MutS homolog 6 (MSH6, diluted 1:300, Cell Signaling Technology, Beverly, MA, USA), and PMS1 homolog 2 (PMS2, diluted 1:40, Cell Marque, Rocklin, CA, USA).

### Analysis of clinical and pathological parameters

All statistical analyses were performed using SPSS software, version 21.0.0.0 for Windows (SPSS Inc., Chicago, IL, USA). To analyze each clinicopathological parameter, the Kruskal-Wallis test, Mann-Whitney test, Fisher's exact test, or the χ2-test were used, depending on the purpose; all *P-*values <0.05 were considered significant. Patient survival statistics were analyzed using the Kaplan-Meier method and log-rank test, and multivariate analyses were performed using the Cox regression model. Hazard ratios (HRs) and corresponding 95% confidence intervals (CIs) are presented.

## SUPPLEMENTARY FIGURES AND TABLES



## Abbreviations

CRC: colorectal cancer
CMS: consensus molecular subtype
MSI: microsatellite instability
NMF: nonnegative matrix factorization
DEG: differentially expressed gene
DFS: disease-free survival
