# Supplementary material for: Prognosis of stage III colorectal carcinomas with FOLFOX adjuvant chemotherapy can be predicted by molecular subtype
Source: Oncotarget. 2017 Apr 11;8(24):39367–81. doi: 10.18632/oncotarget.17023 (PMC5503619; doi:10.18632/oncotarget.17023)
Supplement: Supplementary file 1 [file oncotarget-08-39367-s001.pdf]

Prognosis of stage III colorectal carcinomas with FOLFOX adjuvant chemotherapy can be predicted by molecular subtype

SUPPLEMENTARY FIGURES AND TABLES

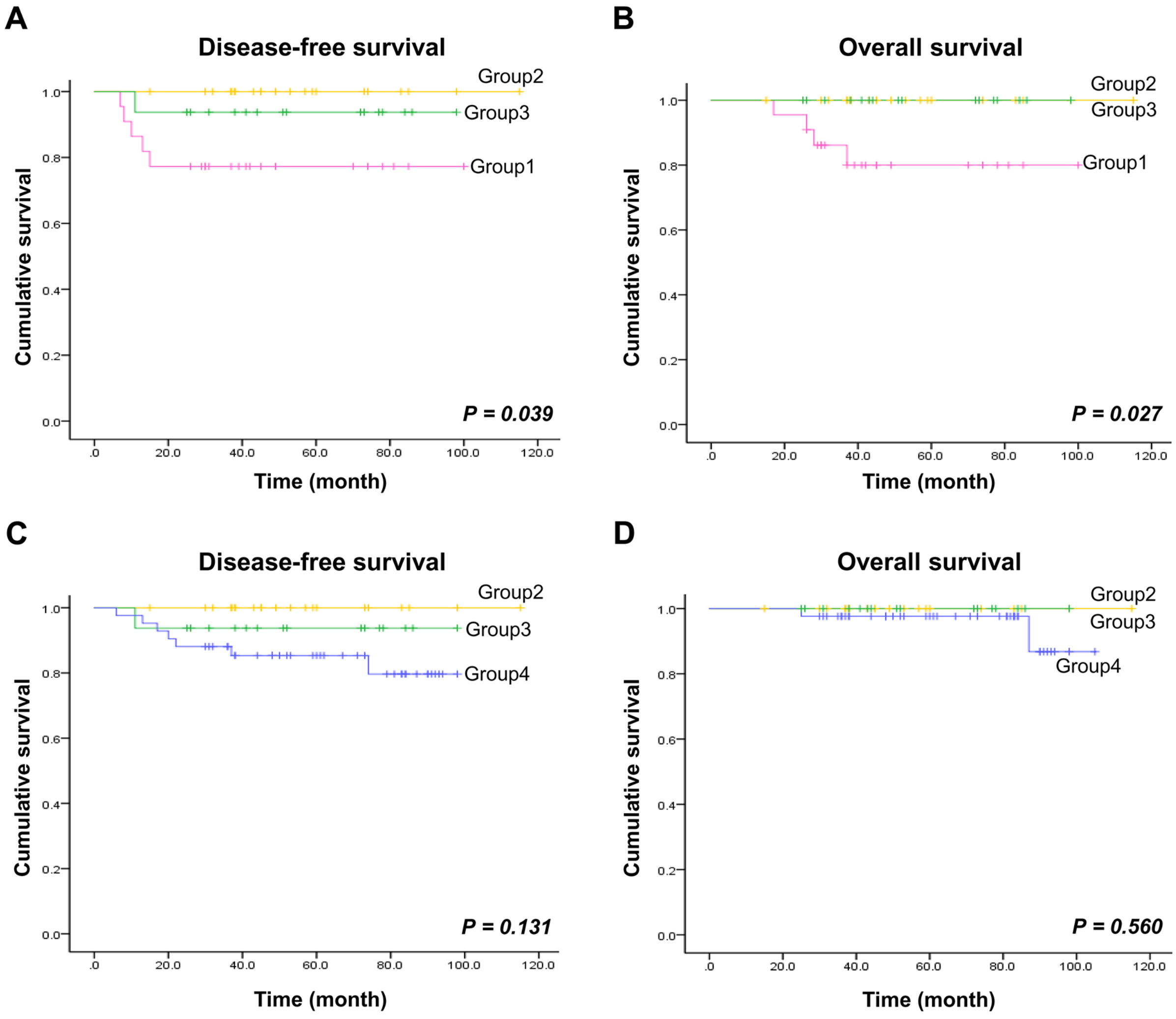

Supplementary Figure 1: Kaplan-Meier survival analysis in CRC molecular subtypes. **(A** and **B)** Disease-free survival and overall survival of groups 1, 2, and 3. **(C** and **D)** Disease-free survival and overall survival of groups 2, 3, and 4.

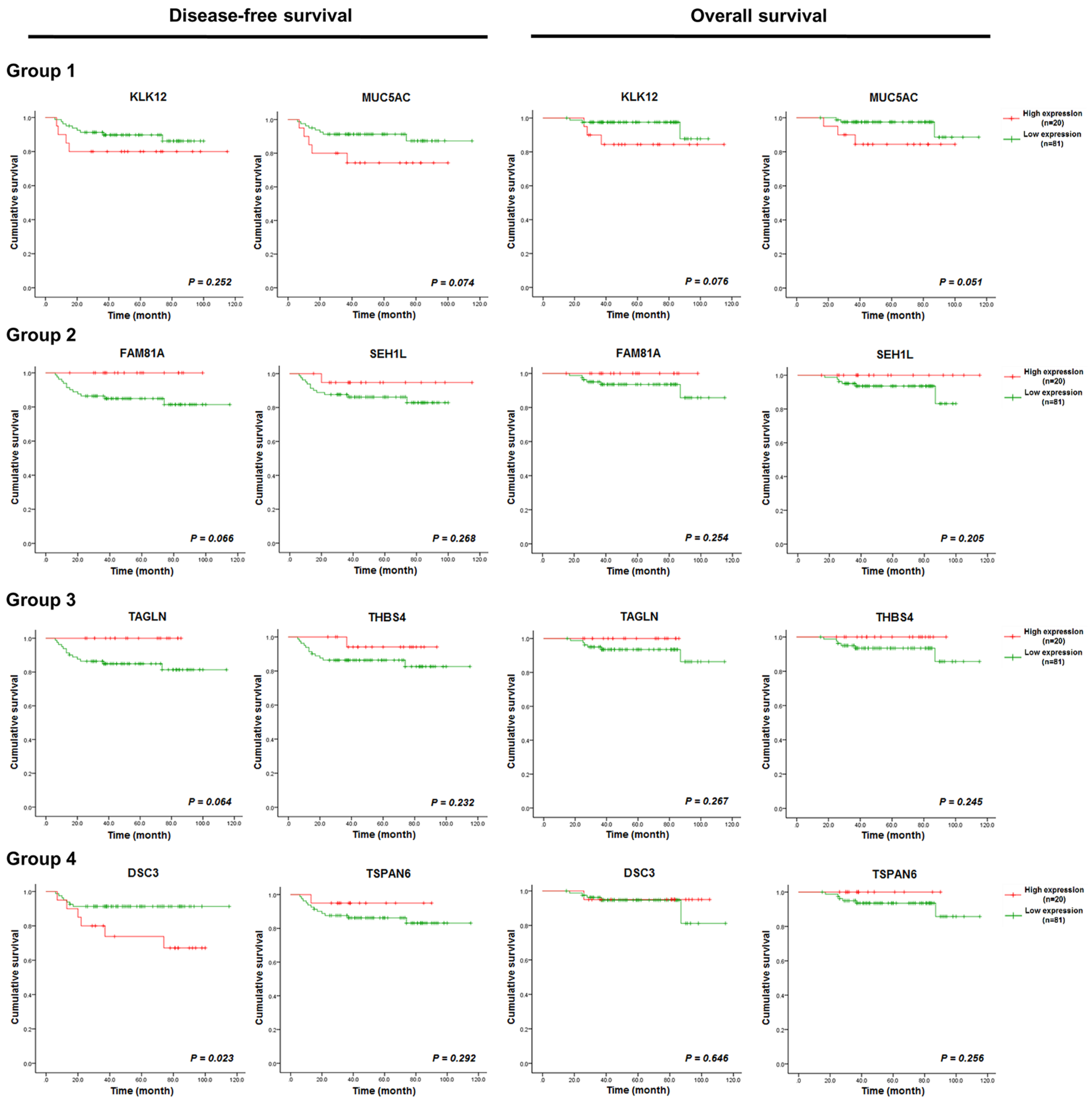

Supplementary Figure 2: Survival curves of patients with stage III CRCs according to the expression of genes representing each CRC subtype. Disease-free survival and overall survival according to the gene expression (n=8). Group 1: *KLK12* and *MUC5AC*; Group 2: *FAM81A* and *SEH1L*; Group 3: *TAGLN* and *THBS4*; Group 4: *DSC3* and *TSPAN6*.

Supplementary Table 1: Concordance rate between subtypes divided by CMS classification and subgroups divided by the gene co-expression network analysis

		Weighted gene co-expression network class				Total
		Group A	Group B	Group C	Group D	
**Predicted CMS class**	CMS1	4 (13.4%)	2 (9.1%)	0 (0%)	4 (20%)	10
	CMS2	13 (43.3%)	14 (63.6%)	5 (62.5%)	6 (30%)	38
	CMS3	3 (10%)	4 (18.2%)	2 (25%)	4 (20%)	13
	CMS4	10 (33.3%)	2 (9.1%)	1 (12.5%)	6 (30%)	19
	undetermined	8	4	2	7	21
	Total	38	26	10	27	101

Supplementary Table 2: Clinicopathologic features of 101 CRCs according to the CMS molecular subtypes

Category	Variables	CMS1 (n=10, 9.9%)		CMS2 (n=38, 37.6%)		CMS3 (n=13, 12.9%)		CMS4 (n=19, 18.8%)		Non consensus (n=21, 20.8%)		*P*-value
Age, years		51.1±15.15		59.08±11.33		61±9.69		60.53±9.82		59.90±10.16		0.465
Gender	male	4	40.0%	22	57.9%	9	69.2%	9	47.4%	7	33.3%	0.234
	female	6	60.0%	16	42.1%	4	30.8%	10	52.6%	14	66.7%	
Location	right side	7	70.0%	6	15.8%	6	46.2%	8	42.1%	9	42.9%	0.008[a]
	left side	3	30.0%	32	84.2%	7	53.8%	11	57.9%	12	57.1%	
Preop CEA level	≤5ng/ml	10	100.0%	26	68.4%	11	84.6%	15	78.9%	11	52.4%	0.046[a]
	>5ng/ml	0	0.0%	12	31.6%	2	15.4%	4	21.1%	10	47.6%	
Gross type	exophytic	10	100.0%	27	71.1%	11	84.6%	14	73.7%	15	71.4%	0.037
	non exophytic	0	0.0%	11	28.9%	2	15.4%	5	26.3%	6	28.6%	
Size		6.36±2.42		4.88±1.66		4.93±2.40		4.62±1.32		5.44±2.26		0.328
Histologic Diagnosis	adeno carcinoma	8	80.0%	38	100.0%	12	92.3%	18	94.7%	19	90.5%	0.014[a]
	mucinous adeno carcinoma	0	0.0%	0	0.0%	1	7.7%	1	5.3%	2	9.5%	
	medullary carcinoma	2	20.0%	0	0.0%	0	0.0%	0	0.0%	0	0.0%	
Mucin formation	absent	4	40.0%	34	89.5%	6	46.2%	17	89.5%	11	52.4%	<.001[a]
	focal	6	60.0%	4	10.5%	6	46.2%	1	5.3%	8	38.1%	
	predominant	0	0.0%	0	0.0%	1	7.7%	1	5.3%	2	9.5%	
Crohn like lymphoid reaction	low-density group	4	40.0%	31	81.6%	7	53.8%	11	57.9%	15	71.4%	0.058
	high-density group	6	60.0%	7	18.4%	6	46.2%	8	42.1%	6	28.6%	
Invasion pattern	expending	5	50.0%	5	13.2%	0	0.0%	2	10.5%	3	14.3%	0.03[a]
	intiltrative	5	50.0%	33	86.8%	13	100.0%	17	89.5%	18	85.7%	
BRAF[b]	Wild type	9	90.0%	38	100.0%	13	100.0%	19	100.0%	21	100.0%	0.099
	Mutation	1	10.0%	0	0.0%	0	0.0%	0	0.0%	0	0.0%	
KRAS[b]	Wild type	8	80.0%	27	71.1%	5	38.5%	13	68.4%	8	38.1%	0.031[a]
	Mutation	2	20.0%	1	28.9%	8	61.5%	6	31.6%	13	61.9%	
MSI status	MSS/ MSI-low	4	40.0%	38	100.0%	12	92.3%	19	100.0%	20	95.2%	0[a]
	MSI high	6	60.0%	0	0.0%	1	7.7%	0	0.0%	1	4.8%	
	MLH1 loss [c]	6		0		0		0		0		
	MSH2 loss [c]	1		0		1		0		0		
	PMS2 loss [c]	5		0		0		0		1		
	MSH6 loss [c]	1		0		1		0		0		

[a]*P*<0.05, [b]Pyrosequencing, [c]Immunohistochemical stain, MSS: Microsatellite stability, MSI: Microsatellite instability
